# Age-Mediated Transcriptomic Changes in Adult Mouse Substantia Nigra

**DOI:** 10.1371/journal.pone.0062456

**Published:** 2013-04-30

**Authors:** Lin Gao, María Hidalgo-Figueroa, Luis M. Escudero, Juan Díaz-Martín, José López-Barneo, Alberto Pascual

**Affiliations:** 1 Instituto de Biomedicina de Sevilla, Hospital Universitario Virgen del Rocío/CSIC/Universidad de Sevilla, Seville, Spain; 2 Centro de Investigación Biomédica en Red sobre Enfermedades Neurodegenerativas, Madrid, Spain; St. Jude Children's Research Hospital, United States of America

## Abstract

Substantia nigra *pars compacta* (SNpc) is highly sensitive to normal aging and selectively degenerates in Parkinson's disease (PD). Until now, molecular mechanisms behind SNpc aging have not been fully investigated using high throughput techniques. Here, we show early signs of aging in SNpc, which are more evident than in ventral tegmental area (VTA), a region adjacent to SNpc but less affected in PD. Aging-associated early changes in transcriptome were investigated comparing late middle-aged (18 months old) to young (2 months old) mice in both SNpc and VTA. A meta-analysis of published microarray studies allowed us to generate a common “transcriptional signature” of the aged (≥ 24 months old) mouse brain. SNpc of late-middle aged mice shared characteristics with the transcriptional signature, suggesting an accelerated aging in SNpc. Age-dependent changes in gene expression specific to SNpc were also observed, which were related to neuronal functions and inflammation. Future studies could greatly help determine the contribution of these changes to SNpc aging. These data help understand the processes underlying SNpc aging and their potential contribution to age-related disorders like PD.

## Introduction

Brain aging is a slow and progressive process, which results in impairment of memory, cognitive and motor functions, and eventually, cell death. In addition, aging is the major risk factor for neurodegenerative diseases, such as Alzheimer's disease (AD) and Parkinson's disease (PD). During the aging process, increases in oxidative, metabolic, and ionic stresses result in neuronal dysfunction and cell death [1].

Different brain regions age differently depending on their specific structure and function. Dopaminergic (DA) neurons in the substantia nigra *pars compacta* (SNpc) are highly sensitive to aging and are progressively and selectively degenerated in PD. Loss of SNpc DA neurons in the elderly occurs at a rate of ∼5% per decade [2]–[4] and is associated with declined motor function [5], [6]. Accelerated aging in SNpc has been supposed to be a consequence of the particular metabolic and electrophysiological properties of DA neurons in this region [7]–[10]. Gene expression profiling of DA neurons in SNpc of elderly individuals indicates an up-regulation of cAMP response element-binding (CREB) and retinoic acid receptors (RAR)-mediated signaling and neuronal function, probably functioning as a compensatory response to aging [11]. In addition, studies on human and rodent individual genes have demonstrated the implication of inflammation [12], iron metabolism [13], DA neuronal function [14]–[22], and vascularization [23] in SNpc aging.

Recent advances in global-genome expression techniques provide a bona fide way to study molecular changes underlying brain aging in both human and rodents [11], [24]–[32]. However, until now molecular mechanisms underlying SNpc aging have not been addressed experimentally in rodent models using high throughput techniques. Therefore, the goal of the current study was to investigate changes in gene expression in SNpc during early aging. Late middle-aged mice (18 months old) without major behavioral alteration were compared with young (2 months old) animals. Side-by-side global gene expression analyses of SNpc and ventral tegmental area (VTA), a DA region adjacent to SNpc but less affected than SNpc in PD, were carried out using microarray techniques. A meta-analysis of published microarray studies resulted in the generation of a “transcriptional signature”, a list of common age-associated genes in aged (≥ 24 months old) mouse brain. In comparison with this transcriptional signature, accelerated aging was suggested in SNpc of late middle-aged mice.

## Materials and Methods

### Animals and Ethics Statement

Wild-type C57BL/6 mice were housed at regulated temperature (22±1°C) in a 12 h light/dark cycle with *ad libitum* access to food and drink. All experiments were performed according to guidelines from the European Community (Council Directive 86/609/EEC) and were approved by the ethical committee of the “Hospital Universitario Virgen del Rocío”. All efforts were made to minimize suffering. Mean life span of C57BL/6 mice are around 26 to 30 months [33], [34]. Three age groups of mice were used in microarray analysis: young (2 months old), middle aged (10 months old), and late-middle aged (18 months old) mice. Four replicates were included in each age group and each replicate was pooled from either 4 or 5 mice (SNpc: 4 mice/replicate×4 replicates×3 age groups; VTA: 5 mice/replicate×4 replicates×3 age groups). Mice were deeply anesthetized by intraperitoneal injection of sodium thiopental before decapitation, followed by brain extraction. Brains were further processed for either immunohistochemical analyses or gene expression studies using microarray or real-time quantitative PCR techniques.

### Behavioral Test

To evaluate the motor function, open-field analysis was performed as described previously [35]. Mouse movement in a box of 22.5×22.5 cm floor and 42 cm-high walls was recorded for 60 min using an automatic tracking system (SMART, Panlab) and analyzed using the SMART software (version 2.5.14). Weight of each mouse was measured before the behavioral test.

### SNpc and VTA Dissection, RNA Extraction, Amplification, and Microarray Analysis

Freshly dissected brain was embedded in gelatin. Coronal sections with a thickness of 500 µm were cut with a vibratome (Leica). According to the mouse brain stereotaxic atlas [36], SNpc and VTA (from −2.5 to −4.0 mm of bregma) were dissected manually under a microscope, quickly frozen with liquid nitrogen, and stored at −80°C for total RNA isolation. For each replicate, total RNA was isolated from pooled SNpc (*n* = 4 mice) or VTA (*n* = 5 mice) using RNeasy micro kit (Qiagen) according to the manufacturer's instruction. The quality of RNA was analyzed using Agilent 2100 Bioanalyzer (Agilent). RNA samples with RNA integrity number (RIN) [37] higher than 7 were further processed for microarray analysis.

A total of 50 ng total RNA of each replicate was applied for synthesis, labeling, and hybridization of cRNA using two-cycle target labeling assay (Affymetrix). Gene expression was studied using GeneChip Mouse Genome 430 2.0 Array (Affymetrix) following the manufacturer's instruction. Images were processed with Affymetrix GeneChip Operating Software 1.4.0.036 (SNpc) or GeneChip Command Console 2.0 (VTA).

Statistical analysis of the microarray data was performed using Babelomics software v4.2 (Medina et al., 2010). Raw expression values (CEL files) were reprocessed for background correlation, normalization, and summarization of probe values using the Robust Multi-chip Average (RMA) algorithm [38]. Differentially expressed genes between conditions were determined by LIMMA, a moderated *t*-test oriented to experiments with few biological replicates [39], [40]. *p* values were adjusted to control the false discovery rate (*pFDR*) using the Benjamini and Hochberg method [41]. Gene expression was considered different between groups with *pFDR* <0.05 and fold change >1.3. Microarray raw data was submitted to Gene Expression Omnibus (GEO) database (accession number GSE45045).

Gene ontology, including biological functions and pathways over-represented by the differentially expressed genes, was analyzed using Ingenuity Pathway Analysis (IPA, September 2011 version, Ingenuity system). The significance of pathway and function over-representation was calculated using Fisher's exact test, and *p* values <0.05 were considered significant.

### Microarray Meta-analysis

To determine common changes in gene expression in aged mouse brain, literature searches in National Center for Biotechnology Information (NCBI) PubMed and dataset searches in NCBI gene expression omnibus (GEO) [42] and ArrayExpress (EBI) [43] were conducted to identify studies of gene expression profiling in aged mouse brain. Studies' inclusion criteria were: 1) compared brain or a brain region between young (2–5 months old) and aged (24–30 months old) mice; 2) studied transcripts on a genome-wide base; 3) used Affymetrix gene expression GeneChip Mouse Genome 430 2.0 Array, the same one used in the current study. Four studies met the inclusion criteria, which used either C57BL/6 strain or a hybrid strain of C57BL/6. Either whole brain, brain without cerebellum and brain stem, or neocortex was used in each study. Raw microarray data (CEL files) were obtained from public repositories [26], [44], [45] or authors of the study [46]. To avoid the difference in data processing and statistic analysis among studies, these studies were re-analyzed using the same method that was used to analyze the microarray data of SNpc and VTA. Briefly, raw expression values (CEL files) were reprocessed using the RMA algorithm and differentially expressed genes between conditions were determined by LIMMA.

To generate a common list of age-associated genes in mouse brain, the list of genes with differential expression from each study was imported into IPA. When a gene appeared in at least three of the four lists, that gene was included into a common list, which was named as “transcriptional signature” of the aged mouse brain. Genes with differential expression in SNpc and VTA were compared with this transcriptional signature and the potential difference in transcriptional signature representation between SNpc and VTA was determined by Chi-square test. In addition, biological pathways and functions over-represented by the differentially expressed genes in SNpc and VTA were also compared with those over-represented by the transcriptional signature. The potential difference in over-represented functions of transcriptional signature between SNpc and VTA was evaluated by Kruskal-Wallis one-way analysis of variance on ranks followed by Tukey test. A *p* value of <0.05 was considered significant.

### Immunohistochemistry

To determine whether the correct regions were dissected for gene expression profiling, brain sections after SNpc or VTA dissection were fixed with 4% paraformaldehyde and then incubated with tyrosine hydroxylase (TH) antibody (rabbit polyclonal, 1∶2000 dilution, Novus Biologicals). Immunoreactivity was detected with 3,3-diaminobenzidine (DAB) using Envision^+^ System-HRP (Dako Cytomation) according to the manufacturer’s instruction. Mouse brain stereotaxic atlas [36] was used as the reference to localize SNpc and VTA.

To detect glial fibrillary acidic protein (GFAP) expression, mouse brains were fixed with 4% paraformaldehyde in PBS and paraffin-embedded. Coronal sections with a thickness of 20 µm were cut with microtome (Leica). Immunoreactivity against GFAP antibody (rabbit polyclonal, 1∶5000 dilution, Dako Cytomation) was detected with DAB using Envision^+^ System-HRP (Dako Cytomation) [47]. The same sections were also incubated with TH antibody (mouse monoclonal, 1∶50 dilution, Sigma), followed by the fluorescent secondary antibody Alexa 568 (1∶500 dilution, Invitrogen) in order to localize SNpc and VTA.

### Real-time Quantitative PCR

To determine the SNpc and VTA dissection accuracy, total RNA was isolated from SNpc and VTA of 2 months old mice. SNpc or VTA from 5 mice were pooled for each replicate. cRNA was amplified using Ambion WT Expression Kit (Life Technologies). 20 ng cRNA was copied to cDNA using QuantiTect Reverse Transcription Kit (Qiagen) in a final volume of 20 µl. Real-time quantitative PCR reactions were performed in a 7500 Fast Real Time PCR System (Life Technologies). PCR reactions were performed in duplicates in a total volume of 20 µl containing 1 µl of cDNA solution and 1 µl of *Calb1* Taqman probe (Life Technologies). A*ctb* was also estimated in each sample to normalize the amount of cRNA input in order to perform relative quantifications.

To validate the microarray results, 20 ng cRNA after the first amplification cycle of cRNA synthesis for the microarray analysis was copied to cDNA using Ambion WT Expression Kit (Life Technologies) in a final volume of 20 µl. Real-time quantitative PCR reactions were performed in a 7500 Fast Real Time PCR System (Life Technologies). PCR reactions were performed in duplicates in a total volume of 20 µl containing 1 µl of cDNA solution and 1 µl of Taqman probe of the specific gene (Life Technologies). *Actb* was also estimated in each sample to normalize the amount of total RNA input in order to perform relative quantifications. In addition, an independent cohort of animals was also used to validate the microarray results following the same procedures explained above.

### Allen Mouse Brain Atlas searching

Gene expression pattern in mouse brain was searched in the Allen Mouse Brain Atlas website (Seattle, Washington: Allen Institute for Brain Science, http://mouse.brain-map.org) [48]. Genes with age-dependent changes in expression in SNpc were searched in the atlas to determine whether their expression was enriched in SNpc in comparison to other brain nuclei. Images from two months old animals were used for the analysis.

### Statistical Analysis

For behavioral test and real-time quantitative PCR, data were presented as mean ± standard error of the mean (s.e.m.). Real-time quantitative PCR was analyzed with Student’s *t*-test. Kolmogorov-Smirnov test was first performed to test whether the data distribution of behavioral analysis was normal and student's *t*-test was used to detect potential difference between groups. Linear regression was applied to determine whether the behavioral change was dependent on weight in old animals. A *p* value of <0.05 was considered significant. Statistical analyses of microarray studies and meta-analysis were described in their corresponding sections.

## Results

### Motor Performance of Late Middle-aged Mice

Motor behavior was first examined in 18 months old mice, which are considered late middle-aged, to determine whether this is the right age to study early molecular changes during aging. Animal's weight was also measured to avoid confounding factors. Female mice weighed 20.57±1.20 g (2 months old) and 29.13±0.72 g (18 months old), whereas male mice weighed 24.05±0.45 g (2 months old) and 35.60±1.13 g (18 months old). As evaluated by open field analysis, traveled distance was similar between 18 and 2 months old mice, indicating a lack of major change in motor function. Although not significant (*p*<0.05), a trend of decrease in resting time was observed in 18 months old mice. On the other hand, average speed of movement was decreased in the aged group compared with the young one (*p*<0.05). After adjusted by weight, as analyzed by linear regression, the average speed in 18 months old mice was still slower than that in 2 months old mice (*p*<0.05), indicating that subtle changes in motor function had already started to occur in 18 months old mice. Overall, the lack of major change in motor function in 18 months old mice supported the decision of using mice with this age to study initial changes during aging.

### Effect of Age on Gene Expression Profile in SNpc

**Figure 1:**
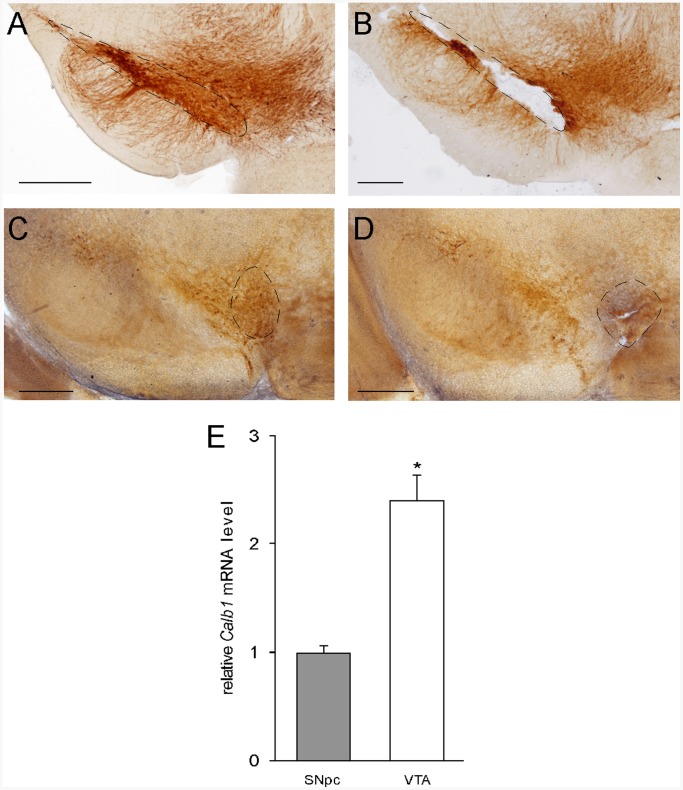
SNpc and VTA dissection from mouse brain. Brain sections were labeled against tyrosine hydroxylase antibody without (**A**) and after (**B**) SNpc dissection, and without (**C**) and after (**D**) VTA dissection. Scale bar: 500 µm. **E**. Relative *Calb1* mRNA level. *, *p*<0.05 comparing VTA to SNpc (*n* = 4 in each group).

In order to study the global effect of aging on gene expression profiles in SNpc and VTA, which depends on the whole cell populations in each nucleus, complete regions instead of DA neurons in each area were dissected from mouse brain. To determine whether the regions were correctly selected, brain sections after the dissection were compared with a section without dissection (Figure 1). As shown by the TH immunoreactivity, which was used to label DA neurons in SNpc and VTA, SNpc was correctly dissected without contamination from VTA (Figure 1B) and vice versa (Figure 1D). High expression of calbindin (*Calb1*) in VTA has been reported compared to SNpc [49]. In agreement with the published data, *Calb1* gene expression was found to be higher from dissected VTA than from SNpc as determined by real-time quantitative PCR (Figure 1E). Both results from immunochemistry and quantitative PCR indicated an accurate SNpc and VTA dissection.

**Figure 2:**
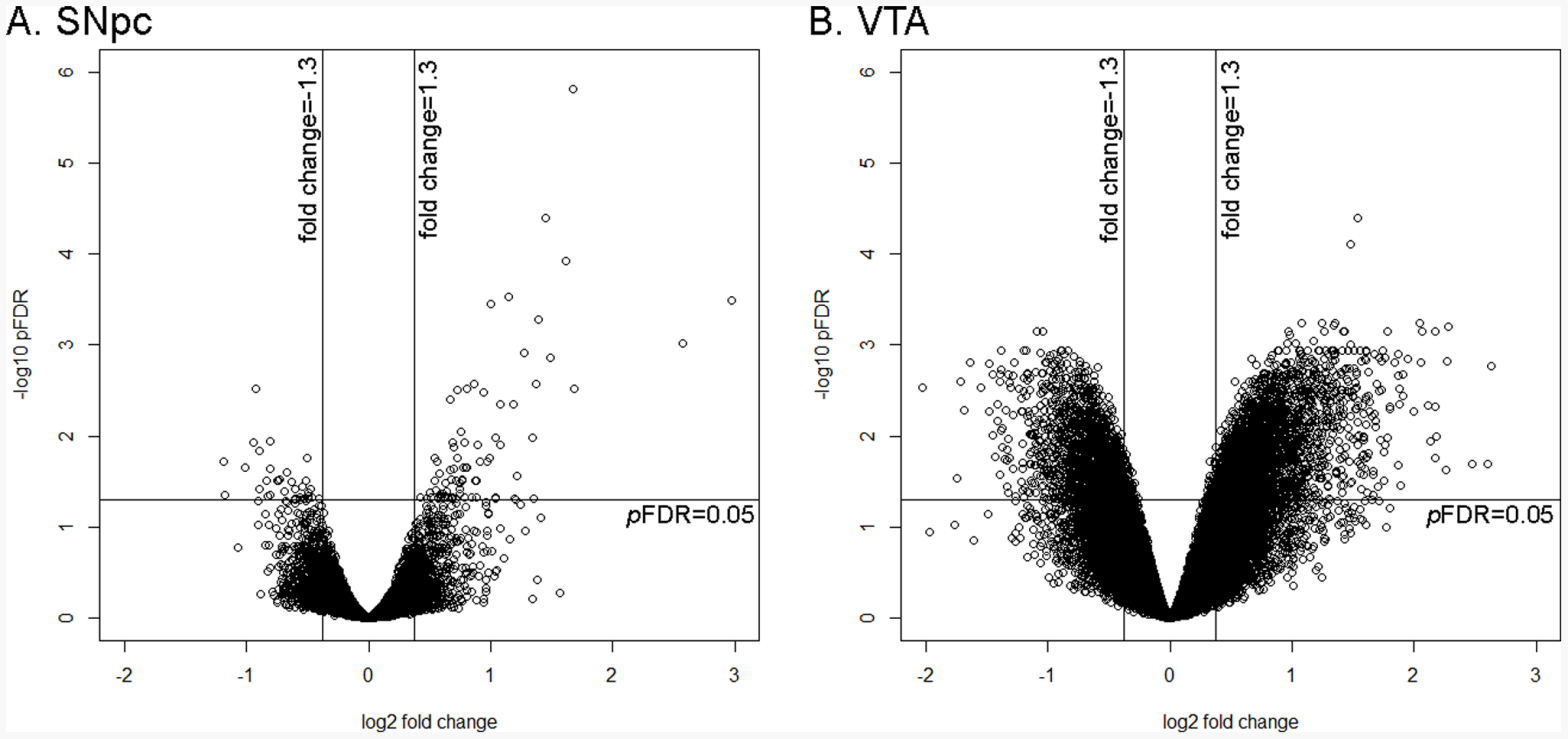
Volcano plots illustrating differential gene expression in SNpc (A) & VTA (B) comparing late middle-aged (18months old) to young (2month old) mice. Differentially expressed genes were distributed in the top left and top right sections of each figure, corresponding *pFDR* <0.05 and fold change<−1.3 and *pFDR* <0.05 and fold change >1.3, respectively.

Microarray analyses were then carried out to study the aging effect on transcriptome in SNpc. GeneChip Mouse Genome 430 2.0 Array (Affymetrix) was used, which is comprised of over 45,000 probe sets and represents over 34,000 mouse genes. As shown in the volcano plot in Figure 2A, differentially expressed genes between 2 and 18 months old mice were distributed in the top left (*pFDR* <0.05 and fold change<-1.3) and top right (*pFDR* <0.05 and fold change >1.3) sections of the plot. Among the 116 differentially expressed probe sets, 99 genes were identified using IPA. These data demonstrated the existence of changes in gene expression profile of mouse SNpc during the early aging process.

**Figure 3:**
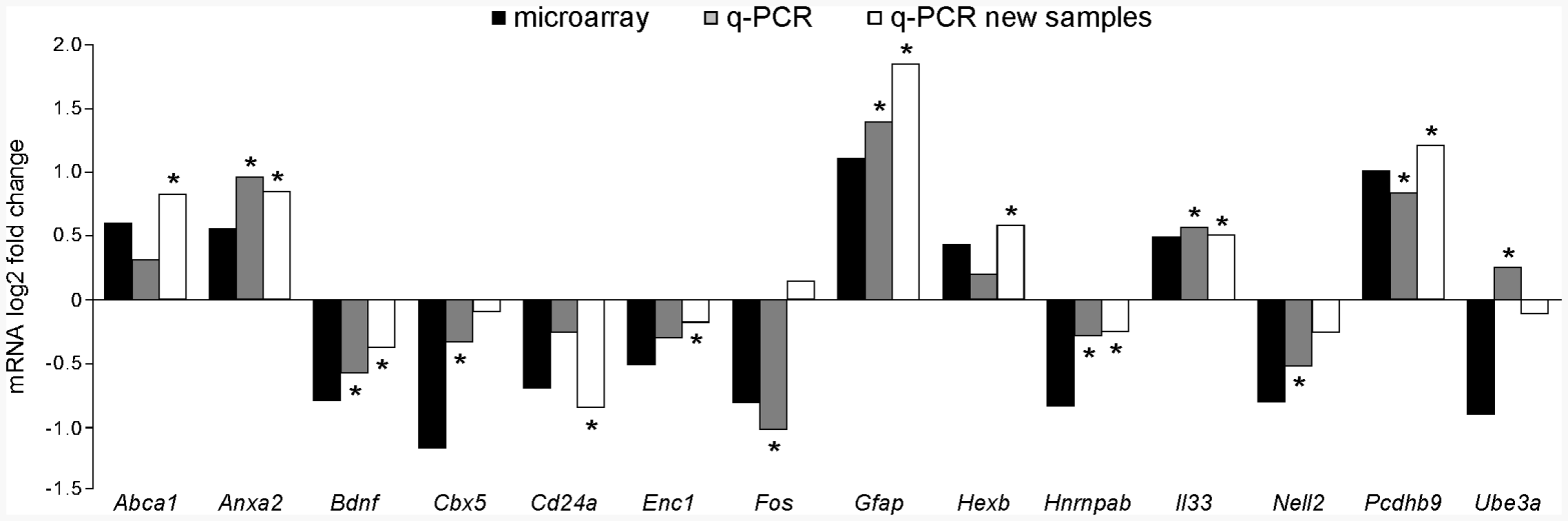
Validation of microarray results in SNpc by real time quantitative PCR. Relative log2 fold changes of mRNA were presented comparing late middle-aged (18months old) to young (2month old) mice. Positive numbers corresponded to up-regulated genes, whereas negative numbers indicated down-regulated genes. *, *p*<0.05 comparing 18 to 2 months old mice using Student’s *t*-test. *n* = 4 replicates for each age group.

Real time quantitative PCR was performed to validate the microarray results. Fourteen genes with the fold change >1.3 and relatively high intensity of raw microarray hybridization signals were selected. Changes in gene expression were confirmed in 9 of 14 genes (64%) using the same RNA samples as microarray (Figure 3). In addition, an independent cohort of mice was also used for the quantitative PCR validation and 10 of 14 genes (71%) were confirmed (Figure 3). Combining the microarray RNA and new RNA samples, 13 of 14 (93%) genes were validated by quantitative PCR.

It was then determined whether the age-dependent genes were expressed in SNpc when compared in the same slices with other adult mice brain nuclei by searching the Allen Mouse Brain Atlas, in which gene expression was detected by *in situ* hybridization. Although quantitative analyses are not possible with this technique, a qualitative comparison was performed. Thirteen of the 99 genes were strongly expressed in SNpc, and 47 of the 99 genes were weakly expressed. Combining the strongly and weakly expressed genes, 60 of 99 genes (61%) were detected by *in situ* hybridization. Expression of the remaining genes was either not detected, possibly due to the technique's limitation on sensibility, or not available (Supplementary Table S1).

To determine the time course of age-dependent changes in gene expression in SNpc, microarray data were also compared between 2 and 10 months old, and 10 and 18 months old mice. Only 3 genes were up-regulated comparing 10 and 2 months groups (3%), which were also increased when comparing 18 and 2 months old groups. No difference in gene expression was detected between 18 and 10 months old groups. These data indicated a gradual age-dependent change in transcriptome in SNpc from 2 to 18 months old mice.

**Figure 4:**
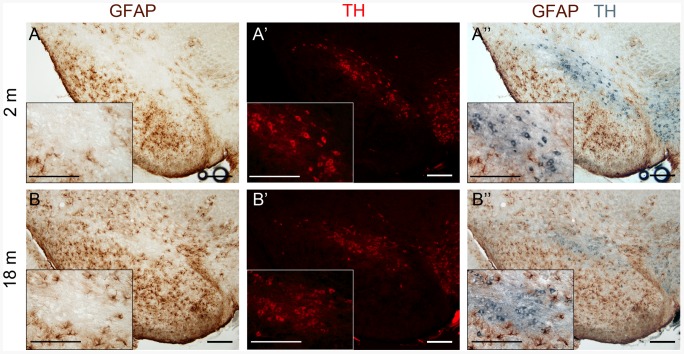
Representative immunohistochemical staining of GFAP in SNpc. **A** and **B**, immuno-staining against GFAP antibody. **A'** and **B'**, immuno-fluorescent staining against TH antibody. **A''** and **B''**, merging of immuno-stainings against GFAP and TH antibodies, the later was represented with a pseudo-color. SNpc was highlighted in each panel. Inset, a higher magnification of SNpc for each picture. Scale bar, 200 µm.

To investigate the nature of genes with differential expression in young and aged mice, gene ontology analysis was carried out using IPA. Top 5 biological functions and canonical pathways were listed in Table 1, indicating that age-dependent genes in mouse SNpc were mainly involved in inflammation, immunological diseases, and cell death. In addition, GFAP immunoreactivity was increased in the SNpc of 18 months old mice (*n* = 3, Figure 4), further supporting the occurrence of a glial response in mice of that age.

**Table 1:** Gene ontology analysis of age-dependent genes in SNpc and VTA comparing 18 to 2 months old mice.

Top 5 functions/pathways	*p* value	No. molecules or ratio
**A. SNpc**		
**Molecular and cellular functions**		
Cell-to-cell signaling and interaction	8.21e-12–7.84e-03	36
Cellular movement	1.37e-09–7.73e-03	29
Cellular compromise	1.02e-07–4.51e-03	16
Cellular growth and proliferation	8.62e-07–7.84e-03	34
Cell death	2.19e-06–7.54e-03	30
**Physiological system development and function**		
Immune cell trafficking	1.37e-09–7.54e-03	26
Hematological system development and function	2.62e-08–7.84e-03	38
Tissue morphology	1.01e-06–7.54e-03	24
Renal and urological system development and function	4.39e-06–4.39e-06	5
Tissue development	6.69e-06–7.54e-03	38
**Canonical pathways**		
IL-10 signaling	3.13e-04	4/78 (0.051)
Systemic lupus erythematosus signaling	3.2e-04	6/228 (0.026)
Complement system	4.79e-04	3/35 (0.086)
B cell receptor signaling	5.68e-04	5/156 (0.032)
Dendritic cell maturation	7.85e-04	5/188 (0.027)
**B. VTA**		
**Molecular and cellular functions**		
Post-translational modification	1.03e-19–5.27e-03	399
Cell cycle	4.11e-18–5.44e-03	415
Cellular assembly and organization	1.68e-13–5.31e-03	477
Cellular function and maintenance	1.68e-13–5.31e-03	399
Gene expression	5.37e-13–4.09e-03	599
**Physiological system development and function**		
Tissue development	9.89e-09–4.59e-03	490
Nervous system development and function	2.33e-08–5.27e-03	420
Connective tissue development and function	3.01e-08–3.94e-03	142
Organismal development	4.46e-06–3.94e-03	454
Embryonic development	2.72e-05–3.94e-03	197
**Canonical pathways**		
Molecular mechanisms of cancer	5.45e-10	100/379 (0.264)
Breast cancer regulation by stathmin1	2.04e-08	63/210 (0.300)
Ephrin receptor signaling	3.21e-08	57/200 (0.285)
Production of nitric oxide and reactive oxygen species in macrophages	4.48e-07	51/187 (0.273)
Integrin signaling	5.79e-07	59/210 (0.281)

### Gene Expression Profiling in VTA

Microarray analyses were also carried out to determine the aging effect on transcriptome in VTA, a DA region adjacent to SNpc. The data distribution was shown in Figure 2B. Comparing 18 and 2 months old mice, 4462 probe sets were differently expressed, among which 3675 genes had been identified using IPA (*pFDR* <0.05 and fold change >1.3). Gene ontology analysis indicated that age-dependent genes in VTA were mainly involved in neurological diseases, post-translational modification, cell cycles, as well as nervous system development and function. This pattern is different from that found for genes in SNpc (Table 1).

To determine the time course of age-induced changes in VTA gene expression, microarray data were also compared between 2 and 10, and 10 and 18 months old mice. Gene expression was changed in 985 probe sets (891 genes) from 2 to 10 months old mice (24.2%), whereas only one gene was differentially expressed from 10 to 18 months old mice. Genes differentially expressed between 2 and 10 months old mice were mainly implicated in neurological diseases, tissue development, cell cycle, embryonic and organ development (Supplementary Table S2). Among them, 846 genes were also detected when comparing 2 and 18 months groups. The large number of differentially expressed genes between 2 and 10 months old mice suggests a maturation of VTA during this post-natal adult period.

### Comparison to Transcriptional Signature of the Aged Mouse Brain

We also investigated whether genes differentially expressed in late middle-aged SNpc coincide with those in aged (≥ 24 months) mouse brain. To this end, common changes in gene expression in aged mouse brain were first evaluated by re-analyzing four published microarray studies [26], [44]–[46] using the same method and criteria as in the current study (see **Materials and methods**). By comparing the lists of differentially expressed genes from each study using IPA, a “transcriptional signature” of 61 common age-associated genes, which appeared in at least three of the four studies, was generated (supplementary Table S3). Twenty-four of the 99 (24.2%) genes with differential expression in SNpc of 18 months old mice were present in the transcriptional signature of ≥ 24 months old mouse brain. In contrast, only 36 of the 3675 (0.1%) genes differentially expressed in the 18-month old VTA also appeared in the transcriptional signature. In order to perform a fair comparison between SNpc and VTA, only the top 120 probe sets with the lowest *pFDR* values in VTA were considered, and in this case, 9 of 114 (7.9%) genes were included in the transcriptional signature of aged mouse brain. Comparing the later ratio in VTA to that in SNpc, the transcriptional signature of aged brain (≥ 24 months old) was more represented in SNpc than VTA of 18 months old mice (χ^2^ = 10.82, df = 1, *p*<0.05).

**Figure 5:**
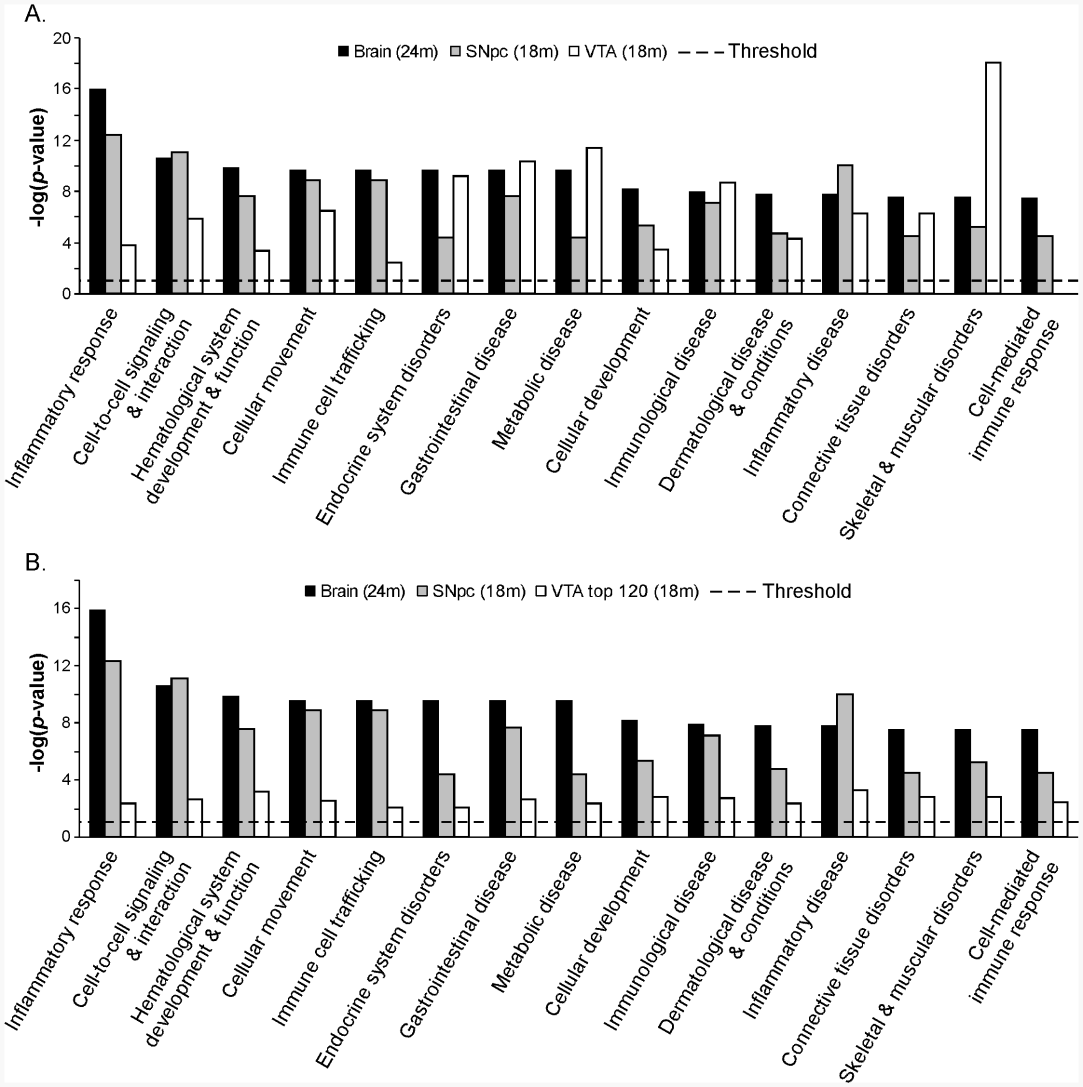
Top 15 biological functions over-represented in aged (≥ 24 months old) mouse brain in comparison to SNpc and VTA in late middle-aged (18 months old) mice. **A**. VTA; **B**. Top 120 probe sets with the lowest *pFDR* values in VTA. Threshold: *p = *0.05.

Biological functions over-represented by the transcriptional signature of the aged brain were compared with those of SNpc and VTA. As shown in Figure 5A, the top 15 functions over-represented by the transcriptional signature were mainly implicated in inflammatory and immune responses and were also over-represented in SNpc and VTA. However, when only the top 5 functions were considered, there was a similarity between the transcriptional signature and SNpc (*p*>0.05), but not between the signature and VTA (*p*<0.05). When only the top 120 probe sets in VTA were considered (Figure 5B), *p* values of all the top 15 functions were similar between SNpc and signature (*p*>0.05), but not between VTA and the signature (*p*<0.05). These results indicate that gene expression profile in SNpc of 18 months old mice is similar to the transcriptional signature of the ≥ 24 months old mouse brain, suggesting an early aging in SNpc.

### Age-associated Genes Specific to SNpc

A list of age-associated genes specific to SNpc was then generated using IPA. Comparing the age-associated genes in SNpc to those in VTA and the transcriptional signature of the aged mouse brain, a list of 43 age-associated genes specific to SNpc was obtained, which were in neither the signature nor the VTA list (Table 2). Using gene ontology analysis, it was found that the top 5 biological functions and canonical pathways over-represented by these genes were mainly related to inflammatory disease, nervous system development and function, cellular growth and proliferation (Table 3). These results suggest that in addition to general brain changes during aging, differential gene expression specific to SNpc in comparison to that of the whole brain of aged mice also occurs. However, the magnitude of the SNpc specific changes was small and further independent validation will be required.

**Table 2:** Age-associated genes specific to mouse SNpc.

Gene symbol	Entrez gene description	Fold change*	*pFDR*
*1600021P15Rik*	RIKEN cDNA 1600021P15 gene	−1.40	0.041
*AA467197*	expressed sequence AA467197	1.60	0.048
*Ang*	angiogenin, ribonuclease, RNase A family, 5	1.61	0.011
*Arhgap30*	Rho GTPase activating protein 30	1.42	0.047
*Arhgap36*	Rho GTPase activating protein 36	−1.59	0.024
*Arhgef37*	Rho guanine nucleotide exchange factor (GEF) 37	1.60	0.046
*Baiap3*	BAI1-associated protein 3	−1.45	0.048
*Bcl2a1a*	B-cell leukemia/lymphoma 2 related protein A1a	1.84	0.030
*Ctbs*	chitobiase, di-N-acetyl-	1.49	0.048
*Cybb*	cytochrome b-245, beta polypeptide	2.53	0.010
*Cyp1b1*	cytochrome P450, family 1, subfamily B, polypeptide 1	1.56	0.040
*Cyp51*	cytochrome P450, family 51	−1.38	0.047
*Defb7*	defensin beta 7	1,99	0.017
*Efemp1*	epidermal growth factor-containing fibulin-like extracellular matrix protein 1	1.73	0.047
*Egr1*	early growth response 1	−1.90	0.003
*Ehbp1l1*	EH domain binding protein 1-like 1	−1.39	0.038
*Emr1*	egf-like module containing, mucin-like, hormone receptor-like sequence 1	1.61	0.046
*Enc1*	ectodermal-neural cortex 1	−1.43	0.017
*Fam70a*	family with sequence similarity 70, member A	−1.46	0.036
*Fcgr3*	Cd16, Fc gamma 3R Alpha, Fc Gamma R3, Fc Gamma R3 Alpha, Fcgr3a	1.46	0.030
*Fos*	FBJ osteosarcoma oncogene	−1.75	0.022
*Gamt*	guanidinoacetatemethyltransferase	−1.51	0.037
*Gcnt1*	glucosaminyl (N-acetyl) transferase 1, core 2	1.60	0.029
*Glis3*	GLIS family zinc finger 3	1.54	0.045
*Gm16440*	predicted gene 16440	1.65	0.017
*Il2rg*	interleukin 2 receptor, gamma chain	1.80	0.047
*Kctd1*	potassium channel tetramerisation domain containing 1	−1.44	0.048
*Lilrb4*	leukocyte immunoglobulin-like receptor, subfamily B, member 4	1.74	0.003
*Naalad2*	N-acetylated alpha-linked acidic dipeptidase 2	1.81	0.002
*Ncf1*	neutrophil cytosolic factor 1	1.54	0.047
*Nell2*	NEL-like 2 (chicken)	−1.74	0.044
*Nupr1*	nuclear protein 1	1.42	0.041
*Pdzrn3*	PDZ domain containing ring finger 3	−1.47	0.049
*Prkg1*	protein kinase, cGMP-dependent, type I	−1.67	0.030
*Pth2*	parathyroid hormone 2	−1.67	0.030
*Rprm*	reprimo, TP53 dependent G2 arrest mediator candidate	−1.55	0.031
*Serping1*	serine (or cysteine) peptidase inhibitor, clade G, member 1	1.46	0.038
*Shisa3*	shisa homolog 3 (Xenopus laevis)	−1.86	0.014
*Tceal5*	transcription elongation factor A (SII)-like 5	−1.51	0.048
*Tmeff2*	transmembrane protein with EGF-like and two follistatin-like domains 2	−1.32	0.048
*Txlnb*	taxilin beta	1.53	0.042
*Ube3a*	ubiquitin protein ligase E3A	−1.86	0.038
*Zbtb26*	zinc finger and BTB domain containing 26	−1.40	0.048

* Comparing 18 months old to 2 months old mice, positive numbers corresponded to up-regulated genes, whereas negative numbers indicated down-regulated genes.

**Table 3:** Gene ontology analysis of age-associated genes specific to SNpc.

Top 5 functions/pathways	*p* value	No. molecules or ratio
**Molecular and cellular functions**		
Cell-to-cell signaling and interaction	6.17e-05–3.44e-02	14
Cellular growth and proliferation	1.09e-04–4.15e-02	16
Free radical scavenging	3.87e-04–3.45e-02	3
Gene expression	4.92e-04–4.15e-02	6
Cellular movement	5.74e-04–3.97e-02	13
**Physiological system development and function**		
Nervous system development and function	3.26e-05–3.62e-02	9
Hematological system development and function	3.76e-04–4.15e-02	11
Humoral immune response	3.76e-04–3.79e-02	7
Tissue morphology	3.76e-04–3.44e-02	10
Immune cell trafficking	6.80e-04–3.97e-02	8
**Canonical pathways**		
B cell receptor signaling	2.55e-03	3/156 (0.019)
IL-2 signaling	4.58e-03	2/58 (0.034)
IL-10 signaling	7.73e-03	2/78 (0.026)
Linoleic acid metabolism	1.11e-02	2/108 (0.019)
Metabolism of xenobiotics by cytochrome P450	1.92e-02	2/197 (0.010)

## Discussion

Changes in transcriptome during the initial stage of brain aging have not been fully addressed. Information about early SNpc aging, a region selectively degenerated in PD, is also very scant. Therefore, we have studied gene expression profile in mouse SNpc during the initial stage of aging in comparison with that in VTA from mice of the same age, and with the published data of aged mouse brain as well. Human studies of frontal cortex suggest that aging starts in young adult life (middle-aged people >40 years old) [28]. A progressive decline in locomotor activity, as determined by open field analysis, has been observed previously from 6 to 24 months old mice [24]. In the current study, 18 months old mice lacking major changes in locomotor activity was used to avoid confusing factors like dramatic cell death and pathological changes associated with advanced aging. Whole SNpc or VTA regions instead of dispersed DA neurons was analyzed, which allowed us to study in each region not only the contribution from DA neurons, but also from non-DA neurons and glia cells. The genes related to inflammation demonstrated the contributions of glia/microglia. On the other hand, neuronal gene expression was also modified and some genes were up-regulated (Table 2), demonstrating the contribution of neurons. The results demonstrated that even in late middle-aged mice, there are changes in gene expression suggesting premature aging in SNpc.

### Effect of Age on Gene Expression Profile in SNpc

A total of 99 genes were found to be differentially expressed in SNpc comparing 18 to 2 months old mice. The magnitude of changes in gene expression was relatively small, with the largest fold change less than 6, which is similar to previously published studies [27], [50]. A complex region, containing neurons, astroglia, and other cells were analyzed in our study, which could dilute specific effects of aging on dopaminergic cells. Therefore, a 1.3 fold change in the whole SNpc region could result from a fold change much higher than 1.3 in dopaminergic neurons. Despite of the relatively small magnitude of changes, 13 of 14 genes were validated using real time quantitative PCR including those with small fold of changes such as *Hexb*, *Il33*, and *Enc1* (Figure 3). The qualitative study of gene expression pattern in mouse brain (Allen Brain Atlas) also supported the microarray results (Supplementary Table S1). Furthermore, 48 of 99 genes (48%) with altered expression in SNpc were also differentially expressed in VTA, a neighboring region with a similar cell population to SNpc. Twenty-four of 99 (24.2%) genes were also over-presented in aged mouse brain (see discussion below). Taken together, these data validated the microarray results.

### Gene Expression Profiling in VTA

Comparing 2 and 18 months old mice, 3675 genes were differently expressed in VTA, which was much higher than that in SNpc (99 genes). A possible explanation of the difference in number of genes could be sample preparation. Due to the limited amount of VTA tissue obtained from each mouse, VTA from 5 mice had to be pooled for each replicate instead of 4 mice for SNpc. The increase in the number of mice used in each replicate of VTA could decrease the variability among replicates, leading to the detection of high number of differentially expressed genes when compared between groups. However, the magnitude of changes (fold change) in gene expression in SNpc was similar to that in VTA (Figure 2), indicating that the results of differentially expressed genes in SNpc are reliable, although it is possible that the number of differentially expressed genes in SNpc could be underestimated.

Intrinsic properties of each brain nuclei could also contribute as a major cause of difference in the number of differentially expressed genes found between SNpc and VTA. Indeed, different brain regions age at different speed and respond to aging with different molecular changes [25], [27], [32]. Unlike SNpc, in which gene expression was barely changed from 2 to 10 months (3%), changes in expression of 891 genes (24%) in VTA were detected during the same period. Gene ontology analysis suggested that these genes were related to tissue and organ development, cell growth, and neuronal function (Supplementary Table S2), suggesting a postnatal maturation of VTA and/or high plasticity associated with VTA function. Specifically, TH, monoamine oxidase B, and quinoid dihydropteridine reductase, which are involved in DA receptor signaling pathway, were up-regulated. Presenilin 1 (PSEN1, a component of γ-secretase complex) and calpain were also up-regulated, whereas β-secretase (BACE1) was down-regulated. These enzymes are involved in the amyloid-processing pathway. The decrease in β-secretase mRNA points to a protective role against amyloid-mediated toxic effect in VTA that could help to explain the resistance of this brain region in neurodegenerative disorders. Recently, presenilin 1 has been shown to be necessary for lysosomal proteolysis and autophagy function, reinforcing a protective role of presenilin 1 induction in VTA aging [51].

### Comparison to Transcriptional Signature of the Aged Mouse Brain: Gene List and Pathway Analysis

To our knowledge, no study about the effect of age on transcriptome in mouse SNpc has been published, which makes it impossible to compare our data with other mouse SNpc studies. On the other hand, we hypothesized that at least some changes should be common to all brain regions including SNpc. In order to test this hypothesis, a list of 61 common age-associated genes (transcriptional signature) in ≥ 24 months old mouse brain was first generated (Table S3) and compared with the SNpc data. The transcriptional signature was obtained by re-analyzing four previously published microarray studies of aged mouse brain (whole brain, brain without cerebellum and brain stem, or neocortex) [26], [44]–[46]. A meta-analysis of 4 studies is considered to provide reliable information of common changes during aging, since 3 to 8 studies have been used in similar meta-analyses [25], [26]. In addition, the variability was minimized using strict criteria to select studies. VTA data from late middle-aged mice were used as the reference data of early aging point to compare with SNpc, since no sufficient published data were available to generate another list of common age-associated genes in 18 months old mouse brain. The top 120 probe sets with the lowest *pFDR* values in VTA were used to obtain a reasonable comparison between SNpc and VTA since the number of differentially expressed genes was different between SNpc and VTA. Genes in the transcriptional signature of ≥ 24 months old mouse brain were more represented in SNpc than in VTA of 18 months old mice, suggesting an early aging in mouse SNpc. Some caution is needed when comparing the aging effect on SNpc and VTA due to the differential post-natal development in these regions.

Although comparison of gene lists has been commonly used to analyze microarray data, pathway and function comparison could give extra or even more accurate information than gene list comparison since different genes being differentially expressed could affect the same pathway or biological function [11], [52]. In agreement with the gene list analysis, comparison analysis of pathways and functions also showed a higher similarity between ≥ 24 months old mouse brain and SNpc from 18 months old mice than that between ≥ 24 months old mouse brain and VTA from 18 months old mice (Figure 5), further supporting the existence of an early aging in mouse SNpc. Inflammatory response is highly represented by the transcription signature in ≥ 24 months old mouse brain (Figure 5). The increased GFAP immunoreactivity also supported the occurrence of inflammatory response in SNpc of 18 months old mice (Figure 4). Although increased GFAP immunoreactivity was also observed in other brain regions including VTA, no changes in GFAP mRNA level in VTA was detected in the current microarray study. A possible explanation is that the low levels of GFAP, as shown by immunoreactivity in Figure 4, in SNpc of 2 months old mice made it easier to detect any changes statistically. An increase in GFAP expression is also observed in VTA of 18 months old mice (Figure 4). However, these differences did not reach statistical significance in our mRNA study probably indicating a change of smaller magnitude.

### Age-associated Genes Specific to SNpc

In the current study, 43 age-associated genes were found to be specific to SNpc (Table 2). Several biological functions over-represented by these genes were also over-represented by the transcriptional signature of ≥ 24 months old mouse brain. Whether the effect of SNpc-specific genes on these functions is similar to that of transcriptional signature remains to be examined in future studies. On the other hand, the transcriptional signature could be under-estimated, since only genes that were found to be differentially expressed in at least three of four studies were included. Therefore, it could not be ruled out that some of these genes were also affected by age in other brain nuclei. Future studies to replicate the current work not only including SNpc, VTA, but also cortex could help clarify these doubts.

### Conclusion

Our results suggest an early aging in mouse SNpc using microarray techniques and comparative analyses of gene lists and biological functions. These changes in SNpc during the initial stage of aging partially overlap general changes observed in aged mouse brain. In addition, changes specific to SNpc are described. Identification of genes that are selectively regulated in the SNpc of middle aged individuals could help to better understand the function and intrinsic properties of SNpc and to explain the selective sensitivity of SNpc to aging and PD.

## Supporting Information

Table S1 **Expression pattern of age-dependent genes in adult mouse SNpc.**
(DOC)

Table S2 **Gene ontology analysis of age-dependent genes in VTA between 2 and 10 months old mice.**
(DOCX)

Table S3 **Transcriptional signature of the aged (≥ 24 months old) mouse brain.**
(DOCX)

## References

[1] Mattson MP, Magnus T (2006) Ageing and neuronal vulnerability. Nature Reviews Neuroscience 7: 278–294. 16552414 10.1038/nrn1886 PMC3710114

[2] Fearnley JM, Lees AJ (1991) Ageing and Parkinson's disease: substantia nigra regional selectivity. Brain 114 (Pt 5): 2283–2301. 10.1093/brain/114.5.2283 1933245

[3] Stark AK, Pakkenberg B (2004) Histological changes of the dopaminergic nigrostriatal system in aging. Cell and Tissue Research 318: 81–92. 15365813 10.1007/s00441-004-0972-9

[4] Rudow G, O'Brien R, Savonenko AV, Resnick SM, Zonderman AB, et al (2008) Morphometry of the human substantia nigra in ageing and Parkinson's disease. Acta Neuropathol 115: 461–470. 18297291 10.1007/s00401-008-0352-8 PMC2431149

[5] Ross GW, Petrovitch H, Abbott RD, Nelson J, Markesbery W, et al (2004) Parkinsonian signs and substantia nigra neuron density in decendents elders without PD. Annals of Neurology 56: 532–539. 15389895 10.1002/ana.20226

[6] Rollo CD (2009) Dopamine and aging: intersecting facets. Neurochemical Research 34: 601–629. 18841466 10.1007/s11064-008-9858-7

[7] Chan CS, Guzman JN, Ilijic E, Mercer JN, Rick C, et al (2007) 'Rejuvenation' protects neurons in mouse models of Parkinson's disease. Nature 447: 1081–1086. 17558391 10.1038/nature05865

[8] Chan CS, Gertler TS, Surmeier DJ (2009) Calcium homeostasis, selective vulnerability and Parkinson's disease. Trends Neurosci 32: 249–256. 19307031 10.1016/j.tins.2009.01.006 PMC4831702

[9] Guzman JN, Sanchez-Padilla J, Wokosin D, Kondapalli J, Ilijic E, et al (2010) Oxidant stress evoked by pacemaking in dopaminergic neurons is attenuated by DJ-1. Nature 468: 696–700. 21068725 10.1038/nature09536 PMC4465557

[10] Mosharov EV, Larsen KE, Kanter E, Phillips KA, Wilson K, et al (2009) Interplay between cytosolic dopamine, calcium, and alpha-synuclein causes selective death of substantia nigra neurons. Neuron 62: 218–229. 19409267 10.1016/j.neuron.2009.01.033 PMC2677560

[11] Elstner M, Morris CM, Heim K, Bender A, Mehta D, et al (2011) Expression analysis of dopaminergic neurons in Parkinson's disease and aging links transcriptional dysregulation of energy metabolism to cell death. Acta Neuropathol 122: 75–86. 21541762 10.1007/s00401-011-0828-9

[12] Villar-Cheda B, Valenzuela R, Rodriguez-Perez AI, Guerra MJ, Labandeira-Garcia JL (2012) Aging-related changes in the nigral angiotensin system enhances proinflammatory and pro-oxidative markers and 6-OHDA-induced dopaminergic degeneration. Neurobiology of Aging 33: 204 e201–204 e211. 10.1016/j.neurobiolaging.2010.08.006 20888078

[13] Zecca L, Stroppolo A, Gatti A, Tampellini D, Toscani M, et al (2004) The role of iron and copper molecules in the neuronal vulnerability of locus coeruleus and substantia nigra during aging. Proc Natl Acad Sci U S A 101: 9843–9848. 15210960 10.1073/pnas.0403495101 PMC470762

[14] Bannon MJ, Poosch MS, Xia Y, Goebel DJ, Cassin B, et al (1992) Dopamine transporter mRNA content in human substantia nigra decreases precipitously with age. Proceedings of the National Academy of Sciences of the United States of America 89: 7095–7099. 1353885 10.1073/pnas.89.15.7095 PMC49652

[15] Bannon MJ, Whitty CJ (1997) Age-related and regional differences in dopamine transporter mRNA expression in human midbrain. Neurology 48: 969–977. 9109886 10.1212/wnl.48.4.969

[16] Chu Y, Kompoliti K, Cochran EJ, Mufson EJ, Kordower JH (2002) Age-related decreases in Nurr1 immunoreactivity in the human substantia nigra. Journal of Comparative Neurology 450: 203–214. 12209851 10.1002/cne.10261

[17] Himi T, Cao M, Mori N (1995) Reduced expression of the molecular markers of dopaminergic neuronal atrophy in the aging rat brain. Journals of Gerontology Series A, Biological Sciences and Medical Sciences 50: B193–200. 10.1093/gerona/50a.4.b193 7614230

[18] Salvatore MF, Pruett BS, Spann SL, Dempsey C (2009) Aging reveals a role for nigral tyrosine hydroxylase ser31 phosphorylation in locomotor activity generation. PLoS One 4: e8466. 20037632 10.1371/journal.pone.0008466 PMC2791868

[19] Schuligoi R, Fernandez J, Heavens RP, Sirinathsinghji DJ (1993) Decreased tyrosine hydroxylase mRNA but not cholecystokinin mRNA in the pars compacta of the substantia nigra and ventral tegmental area of aged rats. Brain Research Molecular Brain Research 19: 333–338. 7901729 10.1016/0169-328x(93)90135-c

[20] Dickerson JW, Hemmerle AM, Numan S, Lundgren KH, Seroogy KB (2009) Decreased expression of ErbB4 and tyrosine hydroxylase mRNA and protein in the ventral midbrain of aged rats. Neuroscience 163: 482–489. 19505538 10.1016/j.neuroscience.2009.06.008 PMC2755587

[21] Chen HL, Lein PJ, Wang JY, Gash D, Hoffer BJ, et al (2003) Expression of bone morphogenetic proteins in the brain during normal aging and in 6-hydroxydopamine-lesioned animals. Brain Research 994: 81–90. 14642451 10.1016/j.brainres.2003.09.020

[22] Castel MN, Beaudet A, Laduron PM (1994) Retrograde axonal transport of neurotensin in rat nigrostriatal dopaminergic neurons. Modulation during ageing and possible physiological role. Biochemical Pharmacology 47: 53–62. 7906122 10.1016/0006-2952(94)90437-5

[23] Villar-Cheda B, Sousa-Ribeiro D, Rodriguez-Pallares J, Rodriguez-Perez AI, Guerra MJ, et al (2009) Aging and sedentarism decrease vascularization and VEGF levels in the rat substantia nigra. Implications for Parkinson's disease. Journal of Cerebral Blood Flow and Metabolism 29: 230–234. 18957989 10.1038/jcbfm.2008.127

[24] Bordner KA, Kitchen RR, Carlyle B, George ED, Mahajan MC, et al (2011) Parallel declines in cognition, motivation, and locomotion in aging mice: association with immune gene upregulation in the medial prefrontal cortex. Experimental Gerontology 46: 643–659. 21453768 10.1016/j.exger.2011.03.003 PMC3664302

[25] Fraser HB, Khaitovich P, Plotkin JB, Paabo S, Eisen MB (2005) Aging and gene expression in the primate brain. PLoS Biology 3: e274. 16048372 10.1371/journal.pbio.0030274 PMC1181540

[26] Kedmi M, Orr-Urtreger A (2011) The effects of aging vs. alpha7 nAChR subunit deficiency on the mouse brain transcriptome: aging beats the deficiency. Age (Dordr) 33: 1–13. 20526689 10.1007/s11357-010-9155-7 PMC3063643

[27] Lee CK, Weindruch R, Prolla TA (2000) Gene-expression profile of the ageing brain in mice. Nat Genet 25: 294–297. 10888876 10.1038/77046

[28] Lu T, Pan Y, Kao SY, Li C, Kohane I, et al (2004) Gene regulation and DNA damage in the ageing human brain. Nature 429: 883–891. 15190254 10.1038/nature02661

[29] Oberdoerffer P, Sinclair DA (2007) The role of nuclear architecture in genomic instability and ageing. Nat Rev Mol Cell Biol 8: 692–702. 17700626 10.1038/nrm2238

[30] Sharman EH, Bondy SC, Sharman KG, Lahiri D, Cotman CW, et al (2007) Effects of melatonin and age on gene expression in mouse CNS using microarray analysis. Neurochemistry International 50: 336–344. 17118492 10.1016/j.neuint.2006.09.001 PMC1868445

[31] Terao A, Apte-Deshpande A, Dousman L, Morairty S, Eynon BP, et al (2002) Immune response gene expression increases in the aging murine hippocampus. Journal of Neuroimmunology 132: 99–112. 12417439 10.1016/s0165-5728(02)00317-x

[32] Xu X, Zhan M, Duan W, Prabhu V, Brenneman R, et al (2007) Gene expression atlas of the mouse central nervous system: impact and interactions of age, energy intake and gender. Genome Biology 8: R234. 17988385 10.1186/gb-2007-8-11-r234 PMC2258177

[33] Lee CK, Klopp RG, Weindruch R, Prolla TA (1999) Gene expression profile of aging and its retardation by caloric restriction. Science 285: 1390–1393. 10464095 10.1126/science.285.5432.1390

[34] Jucker M, Ingram DK (1997) Murine models of brain aging and age-related neurodegenerative diseases. Behavioural Brain Research 85: 1–26. 9095338 10.1016/s0166-4328(96)02243-7

[35] Pascual A, Hidalgo-Figueroa M, Piruat JI, Pintado CO, Gomez-Diaz R, et al (2008) Absolute requirement of GDNF for adult catecholaminergic neuron survival. Nature Neuroscience 11: 755–761. 18536709 10.1038/nn.2136

[36] Franklin KBJ, Paxinos G (2008) The mouse brain in stereotaxic coordinates: Academic Press-Elsevier.

[37] Schroeder A, Mueller O, Stocker S, Salowsky R, Leiber M, et al (2006) The RIN: an RNA integrity number for assigning integrity values to RNA measurements. BMC Mol Biol 7: 3. 16448564 10.1186/1471-2199-7-3 PMC1413964

[38] Irizarry RA, Hobbs B, Collin F, Beazer-Barclay YD, Antonellis KJ, et al (2003) Exploration, normalization, and summaries of high density oligonucleotide array probe level data. Biostatistics 4: 249–264. 12925520 10.1093/biostatistics/4.2.249

[39] Smyth GK (2004) Linear Models and Empirical Bayes Methods for Assessing Differential Expression in Microarray Experiments. Statistical Applications in Genetics and Molecular Biology 3: Article 3. 10.2202/1544-6115.1027 16646809

[40] Jeanmougin M, de Reynies A, Marisa L, Paccard C, Nuel G, et al (2010) Should we abandon the t-test in the analysis of gene expression microarray data: a comparison of variance modeling strategies. PLoS ONE 5: e12336. 20838429 10.1371/journal.pone.0012336 PMC2933223

[41] Benjamini Y, Hochberg Y (1995) Controlling the false discovery rate: a practical and powerful approach to multiple testing. Journal of the Royal Statistical Society Series B (Methodological) 57: 289–300.

[42] Edgar R, Domrachev M, Lash AE (2002) Gene Expression Omnibus: NCBI gene expression and hybridization array data repository. Nucleic Acids Research 30: 207–210. 11752295 10.1093/nar/30.1.207 PMC99122

[43] Parkinson H, Kapushesky M, Shojatalab M, Abeygunawardena N, Coulson R, et al (2007) ArrayExpress–a public database of microarray experiments and gene expression profiles. Nucleic Acids Research 35: D747–750. 17132828 10.1093/nar/gkl995 PMC1716725

[44] Oberdoerffer P, Michan S, McVay M, Mostoslavsky R, Vann J, et al (2008) SIRT1 redistribution on chromatin promotes genomic stability but alters gene expression during aging. Cell 135: 907–918. 19041753 10.1016/j.cell.2008.10.025 PMC2853975

[45] Barger JL, Kayo T, Vann JM, Arias EB, Wang J, et al (2008) A low dose of dietary resveratrol partially mimics caloric restriction and retards aging parameters in mice. PLoS ONE 3: e2264. 18523577 10.1371/journal.pone.0002264 PMC2386967

[46] Perreau VM, Bondy SC, Cotman CW, Sharman KG, Sharman EH (2007) Melatonin treatment in old mice enables a more youthful response to LPS in the brain. Journal of Neuroimmunology 182: 22–31. 17070935 10.1016/j.jneuroim.2006.09.005 PMC1847646

[47] Hidalgo-Figueroa M, Bonilla S, Gutierrez F, Pascual A, Lopez-Barneo J (2012) GDNF Is Predominantly Expressed in the PV+ Neostriatal Interneuronal Ensemble in Normal Mouse and after Injury of the Nigrostriatal Pathway. Journal of Neuroscience 32: 864–872. 22262884 10.1523/JNEUROSCI.2693-11.2012 PMC6621168

[48] Lein ES, Hawrylycz MJ, Ao N, Ayres M, Bensinger A, et al (2007) Genome-wide atlas of gene expression in the adult mouse brain. Nature 445: 168–176. 17151600 10.1038/nature05453

[49] Korotkova TM, Ponomarenko AA, Haas HL, Sergeeva OA (2005) Differential expression of the homeobox gene Pitx3 in midbrain dopaminergic neurons. European Journal of Neuroscience 22: 1287–1293. 16190884 10.1111/j.1460-9568.2005.04327.x

[50] Mirnics K, Pevsner J (2004) Progress in the use of microarray technology to study the neurobiology of disease. Nature Neuroscience 7: 434–439. 15114354 10.1038/nn1230

[51] Lee JH, Yu WH, Kumar A, Lee S, Mohan PS, et al (2010) Lysosomal proteolysis and autophagy require presenilin 1 and are disrupted by Alzheimer-related PS1 mutations. Cell 141: 1146–1158. 20541250 10.1016/j.cell.2010.05.008 PMC3647462

[52] Sutherland GT, Matigian NA, Chalk AM, Anderson MJ, Silburn PA, et al (2009) A cross-study transcriptional analysis of Parkinson's disease. PLoS ONE 4: e4955. 19305504 10.1371/journal.pone.0004955 PMC2654916

