# Supplementary material for: Age-Mediated Transcriptomic Changes in Adult Mouse Substantia Nigra
Source: PLoS One. 2013 Apr 30;8(4):e62456. doi: 10.1371/journal.pone.0062456 (PMC3640071; doi:10.1371/journal.pone.0062456)
Supplement: Table S1 — Expression pattern of age-dependent genes in adult mouse SNpc. (DOC) [file pone.0062456.s001.doc]

**Table S1. Expression pattern of age-dependent genes in adult** mouse SNpc.

| **Gene symbol** | **Entrez gene description** | **Expression pattern*** | **Fold change**** |
| --- | --- | --- | --- |
| *1600021P15Rik* | RIKEN cDNA 1600021P15 gene | SNpc weak / other regions strong | -1.40 |
| *9630013A20Rik* | RIKEN cDNA 9630013A20 gene | not available | -2.28 |
| *A530053G22Rik* | RIKEN cDNA A530053G22 gene | weak general expression | 7.83 |
| *AA467197* | expressed sequence AA467197 | weak general expression | 1.60 |
| *Abca1* | ATP-binding cassette, sub-family A (ABC1), member 1 | weak general expression | 1.51 |
| *Abca8a* | ATP-binding cassette, sub-family A (ABC1), member 8a | not available | 2.22 |
| *Abhd12* | abhydrolase domain containing 12 | strong general expression | -1.40 |
| *Ang* | angiogenin, ribonuclease, RNase A family, 5 | weak general expression | 1.61 |
| *Anxa2* | annexin A2 | SNpc no expression / other regions strong | 1.45 |
| *Anxa4* | annexin A4 | SNpc no expression / other regions strong | 1.51 |
| *Arhgap30* | Rho GTPase activating protein 30 | no expression | 1.42 |
| *Arhgap36* | Rho GTPase activating protein 36 | SNpc weak / other regions strong | -1.59 |
| *Arhgef37* | Rho guanine nucleotide exchange factor (GEF) 37 | weak general expression | 1.60 |
| *Atp10a* | ATPase, class V, type 10A | weak general expression | 1.52 |
| *Baiap3* | BAI1-associated protein 3 | SNpc strong / other regions strong | -1.45 |
| *Bcl2a1a* | B-cell leukemia/lymphoma 2 related protein A1a | weak general expression | 1.84 |
| *Bdnf* | brain derived neurotrophic factor | SNpc strong / other regions strong | -1.75 |
| *C3ar1* | complement component 3a receptor 1 | no expression | 1.62 |
| *C4b* | complement component 4B (Childo blood group) | weak general expression | 3.19 |
| *Casp12* | caspase 12 | weak general expression | 1.85 |
| *Cbx5* | chromobox homolog 5 (Drosophila HP1a) | SNpc weak / other regions weak | -2.27 |
| *Ccl6* | chemokine (C-C motif) ligand 6 | weak general expression | 1.67 |
| *Cd24a* | CD24a antigen | SNpc strong / other regions strong | -1.61 |
| *Cd48* | CD48 antigen | weak general expression | 1.70 |
| *Cd52* | CD52 antigen | weak general expression | 2.28 |
| *Cd68* | CD68 antigen | weak general expression | 1.88 |
| *Clec7a* | C-type lectin domain family 7, member a | no expression | 5.93 |
| *Ctbs* | chitobiase, di-N-acetyl- | no expression | 1.49 |
| *Ctsh* | cathepsin H | no expression | 1.59 |
| *Cybb* | cytochrome b-245, beta polypeptide | no expression | 2.53 |
| *Cyp1b1* | cytochrome P450, family 1, subfamily b, polypeptide 1 | no expression | 1.56 |
| *Cyp2e1* | cytochrome P450, family 2, subfamily e, polypeptide 1 | no expression | 1.86 |
| *Cyp51* | cytochrome P450, family 51 | SNpc strong / other regions strong | -1.38 |
| *Defb7* | defensin beta 7 | not available | 1,99 |
| *Dmp1* | dentin matrix protein 1 | weak general expression | 2.56 |
| *Dpyd* | dihydropyrimidine dehydrogenase | weak general expression | 1.72 |
| *Efemp1* | epidermal growth factor-containing fibulin-like extracellular matrix protein 1 | weak general expression | 1.73 |
| *Egr1* | early growth response 1 | SNpc weak / other regions strong | -1.90 |
| *Ehbp1l1* | EH domain binding protein 1-like 1 | SNpc strong / other regions strong | -1.39 |
| *Emr1* | EGF-like module containing, mucin-like, hormone receptor-like sequence 1 | weak general expression | 1.61 |
| *Enc1* | ectodermal-neural cortex 1 | SNpc no expression / other regions strong | -1.43 |
| *Eya4* | eyes absent 4 homolog (Drosophila) | weak general expression | 3.22 |
| *Fam70a* | family with sequence similarity 70, member A | not available | -1.46 |
| *Fcgr2b* | Fc receptor, IgG, low affinity IIb | weak general expression | 2.42 |
| *Fcgr3* | Fc receptor, IgG, low affinity III | no expression | 1.46 |
| *Fmo2* | flavin containing monooxygenase 2 | no expression | 2.32 |
| *Fos* | FBJ osteosarcoma oncogene | SNpc weak / other regions strong | -1.75 |
| *Gamt* | guanidinoacetate methyltransferase | weak general expression | -1.51 |
| *Gcnt1* | glucosaminyl (N-acetyl) transferase 1, core 2 | SNpc no expression / other regions strong | 1.60 |
| *Gfap* | glial fibrillary acidic protein | weak general expression | 2.29 |
| *Glis3* | GLIS family zinc finger 3 | SNpc weak / other regions strong | 1.54 |
| *Gm16233* | predicted gene 16233 | not available | 2.74 |
| *Gm8267* | predicted gene 8267 | not available | 1.65 |
| *Gpr17* | G protein-coupled receptor 17 | SNpc weak / other regions weak | -1.92 |
| *H2-Q7* | histocompatibility 2, Q region locus 7 | not available | 1.72 |
| *Hexb* | hexosaminidase B | SNpc weak / other regions weak | 1.34 |
| *Hnrnpab* | heterogeneous nuclear ribonucleoprotein A/B | SNpc strong / other regions strong | -1.78 |
| *Il2rg* | interleukin 2 receptor, gamma chain | weak general expression | 1.80 |
| *Il33* | interleukin 33 | weak general expression | 1.39 |
| *Itgb2* | integrin beta 2 | no expression | 1.75 |
| *Kctd1* | potassium channel tetramerisation domain containing 1 | SNpc weak / other regions strong | -1.44 |
| *Lgals3* | lectin, galactose binding, soluble 3 | weak general expression | 2.06 |
| *Lilrb4* | leukocyte immunoglobulin-like receptor, subfamily B, member 4 | weak general expression | 1.74 |
| *Ly86* | lymphocyte antigen 86 | no expression | 1.61 |
| *Lyz1* | lysozyme 1 | weak general expression | 3.06 |
| *Malat1* | metastasis associated lung adenocarcinoma transcript 1 (non-coding RNA) | not available | -2.02 |
| *Mpeg1* | macrophage expressed gene 1 | SNpc weak / other regions weak | 1.96 |
| *Ms4a6d* | membrane-spanning 4-domains, subfamily A, member 6D | weak general expression | 1.63 |
| *Naalad2* | N-acetylated alpha-linked acidic dipeptidase 2 | weak general expression | 1.81 |
| *Ncf1* | neutrophil cytosolic factor 1 | weak general expression | 1.54 |
| *Nell2* | NEL-like 2 (chicken) | SNpc strong / other regions strong | -1.74 |
| *Nupr1* | nuclear protein 1 | no expression | 1.42 |
| *Pcdhb3* | protocadherin beta 3 | no expression | 1.65 |
| *Pcdhb9* | protocadherin beta 9 | weak general expression | 2.00 |
| *Pdzrn3* | PDZ domain containing RING finger 3 | weak general expression | -1.47 |
| *Plek* | pleckstrin | no expression | 1.55 |
| *Plscr1* | phospholipid scramblase 1 | no expression | 1.71 |
| *Pmaip1* | phorbol-12-myristate-13-acetate-induced protein 1 | strong general expression | 1.57 |
| *Prkg1* | protein kinase, cGMP-dependent, type I | SNpc no expression / other regions strong | -1.67 |
| *Pth2* | parathyroid hormone 2 | weak general expression | -1.67 |
| *Ptprc* | protein tyrosine phosphatase, receptor type, C | weak general expression | 2.05 |
| *Ripk4* | receptor-interacting serine-threonine kinase 4 | no expression | 2.06 |
| *Rprm* | reprimo, TP53 dependent G2 arrest mediator candidate | SNpc no expression / other regions strong | -1.55 |
| *Scel* | sciellin | no expression | 1.45 |
| *Serpina3n* | serine (or cysteine) peptidase inhibitor, clade A, member 3N | no expression | 1.69 |
| *Serping1* | serine (or cysteine) peptidase inhibitor, clade G, member 1 | no expression | 1.46 |
| *Shisa3* | shisa homolog 3 (Xenopus laevis) | SNpc no expression / other regions strong | -1.86 |
| *Slc15a3* | solute carrier family 15, member 3 | no expression | 1.68 |
| *Smad6* | MAD homolog 6 (Drosophila) | weak general expression | 1.93 |
| *Tceal5* | transcription elongation factor A (SII)-like 5 | strong general expression | -1.51 |
| *Tmeff2* | transmembrane protein with EGF-like and two follistatin-like domains 2 | strong general expression | -1.32 |
| *Tmem163* | transmembrane protein 163 | SNpc strong / other regions strong | -1.66 |
| *Tspan2* | tetraspanin 2 | weak general expression | -1.48 |
| *Tspo* | translocator protein | no expression | 1.68 |
| *Txlnb* | taxilin beta | strong general expression | 1.53 |
| *Tyrobp* | TYRO protein tyrosine kinase binding protein | weak general expression | 1.89 |
| *Ube3a* | ubiquitin protein ligase E3A | SNpc no expression / other regions weak | -1.86 |
| *Zbtb26* | zinc finger and BTB domain containing 26 | weak general expression | -1.40 |
| *Zc3hav1* | zinc finger CCCH type, antiviral 1 | no expression | 1.59 |

*Based on the data from the Allen Mouse Brain Atlas; **Comparing 18 months old to 2 months old mice, positive numbers corresponded to up-regulated genes, whereas negative numbers indicated down-regulated genes.
