# Supplementary material for: Age-Mediated Transcriptomic Changes in Adult Mouse Substantia Nigra
Source: PLoS One. 2013 Apr 30;8(4):e62456. doi: 10.1371/journal.pone.0062456 (PMC3640071; doi:10.1371/journal.pone.0062456)
Supplement: Table S2 — Gene ontology analysis of age-dependent genes in VTA between 2 and 10 months old mice. (DOCX) [file pone.0062456.s002.docx]

**Table S2: Gene ontology analysis of age-dependent genes in VTA between 2 and 10 months old mice.**

| **Top 5 functions/pathways** | ***p* value** | **No. molecules**  **or ratio** |
| --- | --- | --- |
| **Molecular and cellular functions** |  |  |
| Cell-to-cell signaling and interaction | 3.95e-05 - 3.82e-02 | 58 |
| Cell cycle | 3.75e-05 - 3.82e-02 | 105 |
| Carbohydrate metabolism | 5.42e-05 - 3.82e-02 | 70 |
| Cellular assembly and organization | 6.14e-05 - 3.82e-02 | 120 |
| Cellular growth and proliferation | 2.40e-04 - 2.16e-02 | 129 |
| **Physiological system development and function** |  |  |
| Endocrine system development and function | 3.59e-05 - 3.84e-02 | 11 |
| Tissue development | 3.59e-05 - 3.84e-02 | 140 |
| Embryonic development | 5.44e-05 - 3.84e-02 | 64 |
| Organ development | 5.44e-05 - 3.84e-02 | 33 |
| Organismal development | 5.44e-05 - 3.84e-02 | 129 |
| **Canonical pathways** |  |  |
| Dopamine receptor signaling | 9.22e-04 | 11/95 (0.116) |
| RAR activation | 2.05e-03 | 16/187 (0.086) |
| Corticotropin releasing hormone signaling | 3.38e-03 | 12/136 (0.088) |
| Pyrimidine metabolism | 3.38e-03 | 16/214 (0.075) |
| Amyloid processing | 4.48e-03 | 7/56 (0.125) |
