# Supplementary material for: Age-Mediated Transcriptomic Changes in Adult Mouse Substantia Nigra
Source: PLoS One. 2013 Apr 30;8(4):e62456. doi: 10.1371/journal.pone.0062456 (PMC3640071; doi:10.1371/journal.pone.0062456)
Supplement: Table S3 — Transcriptional signature of the aged (≥ 24 months old) mouse brain. (DOCX) [file pone.0062456.s003.docx]

**Table S3: Transcriptional signature of the aged (≥ 24 months old) mouse brain.**

| **Gene symbol** | **Entrez gene description** |
| --- | --- |
| *Abca8a* | ATP-binding cassette, sub-family A (ABC1), member 8a |
| *Acer2* | alkaline ceramidase 2 |
| *Anln* | anillin, actin binding protein |
| *Apod* | apolipoprotein D |
| *B2m* | beta-2-microglobulin |
| *B3galt5* | UDP-Gal:betaGlcNAc beta 1,3-galactosyltransferase, polypeptide 5 |
| *Bc061194* | cDNA sequence BC061194 |
| *C3* | complement component 3 |
| *C1qa* | complement component 1, q subcomponent, alpha polypeptide |
| *C1qb* | complement component 1, q subcomponent, beta polypeptide |
| *C1qc* | complement component 1, q subcomponent, C chain |
| *C4b* | complement component 4B (Chido blood group) |
| *Ccl6* | chemokine (C-C motif) ligand 6 |
| *Cd52* | CD52 antigen |
| *Cd68* | CD68 antigen |
| *Cd74* | CD74 antigen (invariant polypeptide of major histocompatibility complex, class II antigen-associated) |
| *Clec7a* | C-type lectin domain family 7, member A |
| *Ctss* | cathepsin S |
| *Ctsz* | cathepsin Z |
| *Cyba* | cytochrome b-245, alpha polypeptide |
| *Defb1* | defensin beta 1 |
| *Dmp1* | dentin matrix acidic phosphoprotein 1 |
| *Fcgr2b* | Fc fragment of IgG, low affinity IIb, receptor (CD32) |
| *Gfap* | glial fibrillary acidic protein |
| *Ggta1* | glycoprotein galactosyltransferase alpha 1, 3 |
| *Gpr17* | G protein-coupled receptor 17 |
| *H2-ld* | histocompatibility 2, D region |
| *Hla-b* | major histocompatibility complex, class I, B |
| *Hla-c* | major histocompatibility complex, class I, C |
| *H2-Aa* | histocompatibility 2, class II antigen A, alpha |
| *Ifi27l1* | interferon, alpha-inducible protein 27 like 1 |
| *Ifitm3* | interferon induced transmembrane protein 3 |
| *Il33* | interleukin 33 |
| *Itgb2* | integrin, beta 2 |
| *Lamp2* | lysosomal-associated membrane protein 2 |
| *Lgals3* | lectin, galactoside-binding, soluble, 3 |
| *Lgals3bp* | lectin, galactoside-binding, soluble, 3 binding protein |
| *Loc100044874* | h-2 class I histocompatibility antigen, K-W28 alpha chain-like |
| *Loc100504230* | hypothetical LOC100504230 |
| *Ly86* | lymphocyte antigen 86 |
| *Lyz1* | lysozyme1 |
| *Mpeg1* | macrophage expressed gene 1 |
| *Neat1* | nuclear paraspeckle assembly transcript 1 (non-protein coding) |
| *Osmr* | oncostatin M receptor |
| *Pcdhb3* | protocadherin beta 3 |
| *Pcdhb9* | protocadherin beta 9 |
| *Pisd* | phosphatidylserine decarboxylase |
| *Plek* | pleckstrin |
| *Pmp22* | peripheral myelin protein 22 |
| *Psmb8* | proteasome (prosome, macropain) subunit, beta type, 8 (large multifunctional peptidase 7) |
| *Ptprc* | protein tyrosine phosphatase, receptor type, C |
| *Serpina3* | serine (or cysteine) peptidase inhibitor, clade A, member 3N |
| *Sox11* | SRY-box containing gene 11 |
| *Spp1* | secreted phosphoprotein 1 |
| *Spnb2* | spectrin beta 2 |
| *Trib2* | tribbles homolog 2 (Drosophila) |
| *Trim9* | tripartite motif containing 9 |
| *Tyrobp* | TYRO protein tyrosine kinase binding protein |
| *Vim* | vimentin |
| *Xdh* | xanthine dehydrogenase |
| *Zc3hav1* | zinc finger CCCH-type, antiviral 1 |
